# Author Correction: Discovering the genes mediating the interactions between chronic respiratory diseases in the human interactome

**DOI:** 10.1038/s41467-021-22939-x

**Published:** 2021-04-19

**Authors:** Enrico Maiorino, Seung Han Baek, Feng Guo, Xiaobo Zhou, Parul H. Kothari, Edwin K. Silverman, Albert-László Barabási, Scott T. Weiss, Benjamin A. Raby, Amitabh Sharma

**Affiliations:** 1 Channing Division of Network Medicine, Brigham and Women’s Hospital, Harvard Medical School, Boston, MA USA; 2 Network Science Institute, Center for Complex Network Research, Department of Physics, Northeastern University, Boston, MA USA

Correction to: *Nature Communications* 10.1038/s41467-020-14600-w, published online 10 February 2020.

The original version of this Article omitted a reference to previous work in “Garcia-Vaquero, M.L., Gama-Carvalho, M., Rivas, J.D.L. et al. Searching the overlap between network modules with specific betweenness (S2B) and its application to cross-disease analysis. *Sci. Rep.* **8**, 11555 (2018)”. This has been added as a reference 31 in the second sentence of the third paragraph in Results subsection “Flow centrality between modules.” as follows: “Flow centrality is a betweenness measure that is parametric on a source set and destination set of nodes, and its coverage spans exclusively the shortest paths connecting the two modules, instead of the whole network, similarly to a recently proposed measure called Double Specific Betweenness (S2B) [ref. 31]”. This has been corrected in the PDF and HTML versions of the Article.

