# Supplementary material for: Discovering the genes mediating the interactions between chronic respiratory diseases in the human interactome
Source: Nat Commun. 2020 Feb 10;11:811. doi: 10.1038/s41467-020-14600-w (PMC7010776; doi:10.1038/s41467-020-14600-w)
Supplement: Supplementary file 1 — Supplementary Information [file 41467_2020_14600_MOESM1_ESM.pdf]

Supplementary Information for

Discovering the genes mediating the interactions between chronic respiratory diseases in the human interactome

Maiorino et al.

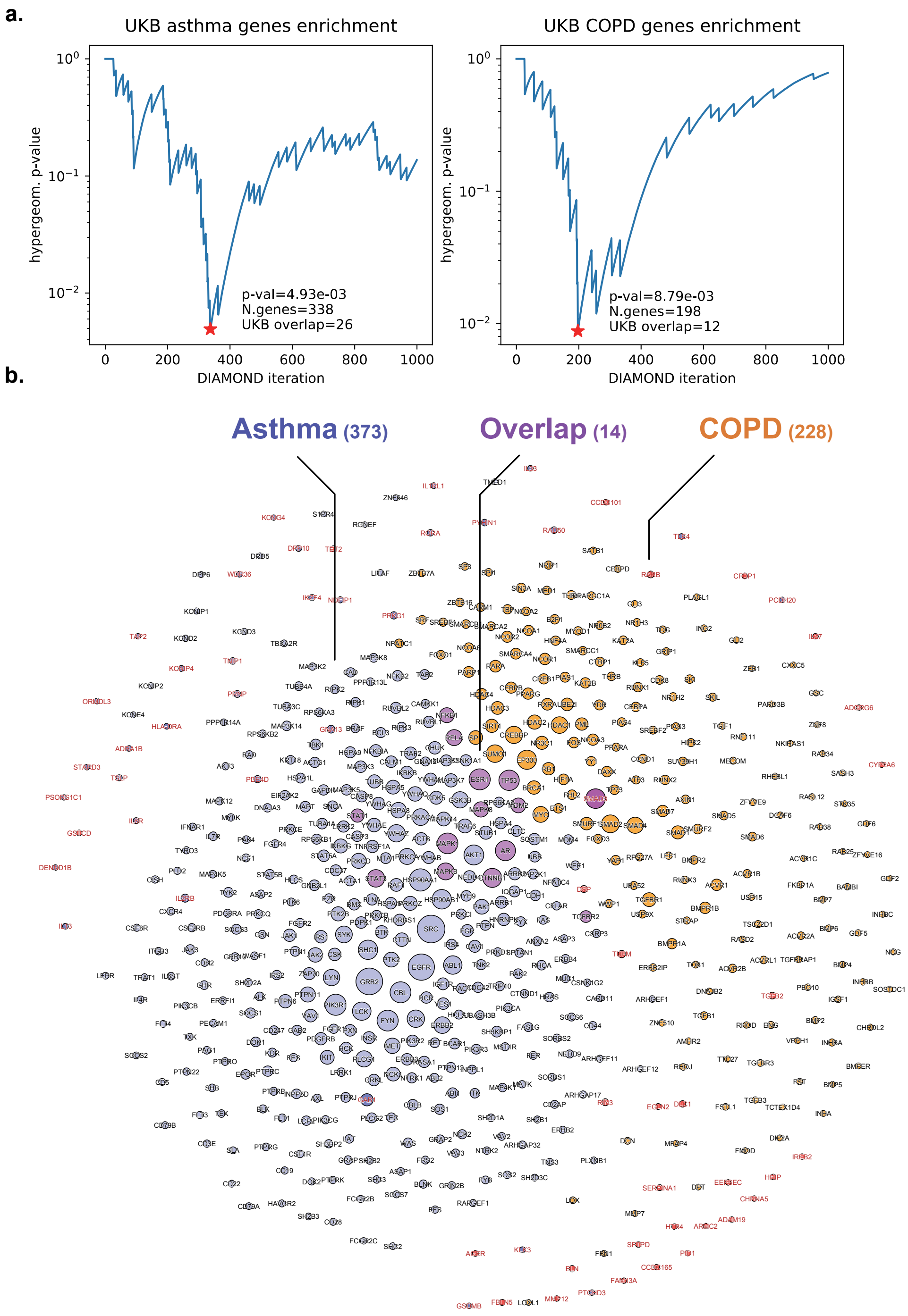

Supplementary Figure 1. (a) Hypergeometric p-value of DIAMOnD module at each iteration for asthma (left) and (COPD) right; (b) Asthma (blue) and COPD (orange) modules, and overlap (purple). Red labels indicate seed genes and node size is proportional to degree.

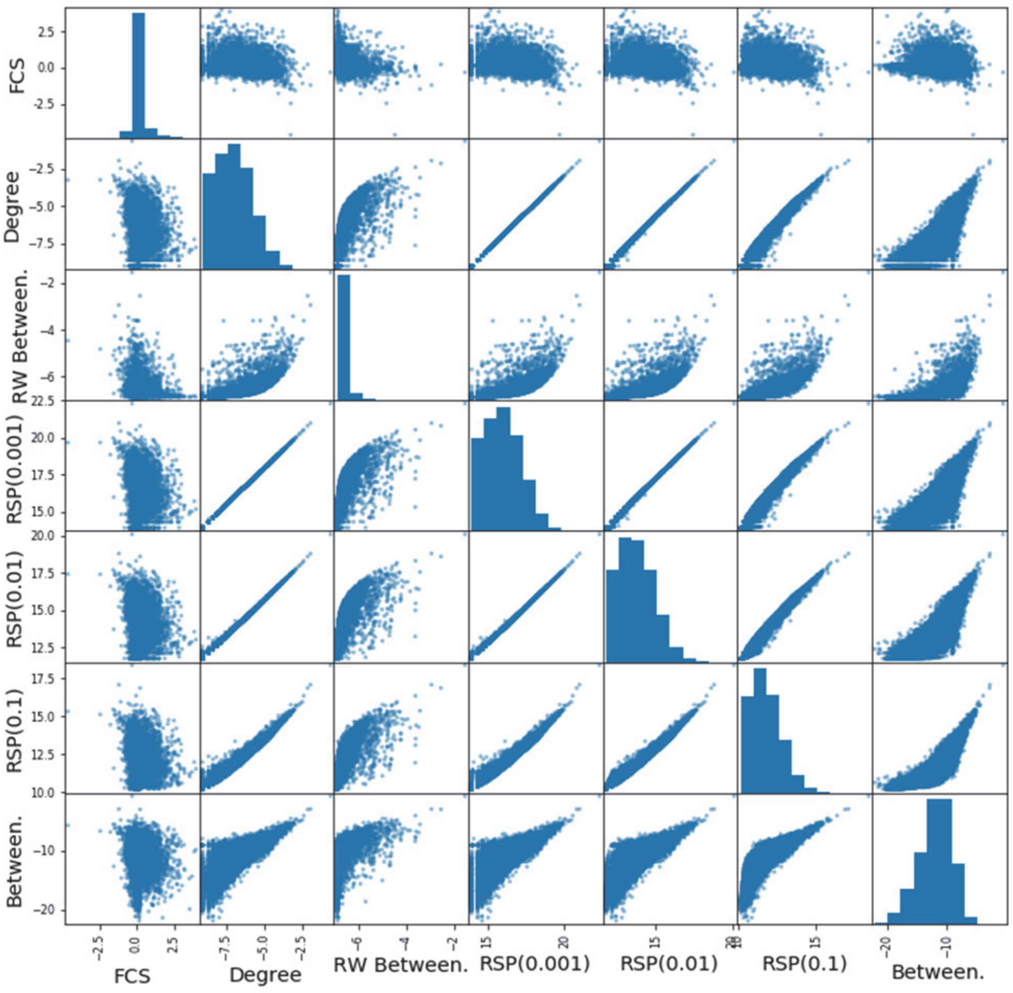

Supplementary Figure 2. Relation of different topological measures: Flow Centrality Score (FCS), Degree, Random Walk betweenness (RW Between.), Randomized Shortest Paths with inverse temperature parameter x (RSP(x)) and shortest-path betweenness

Figure

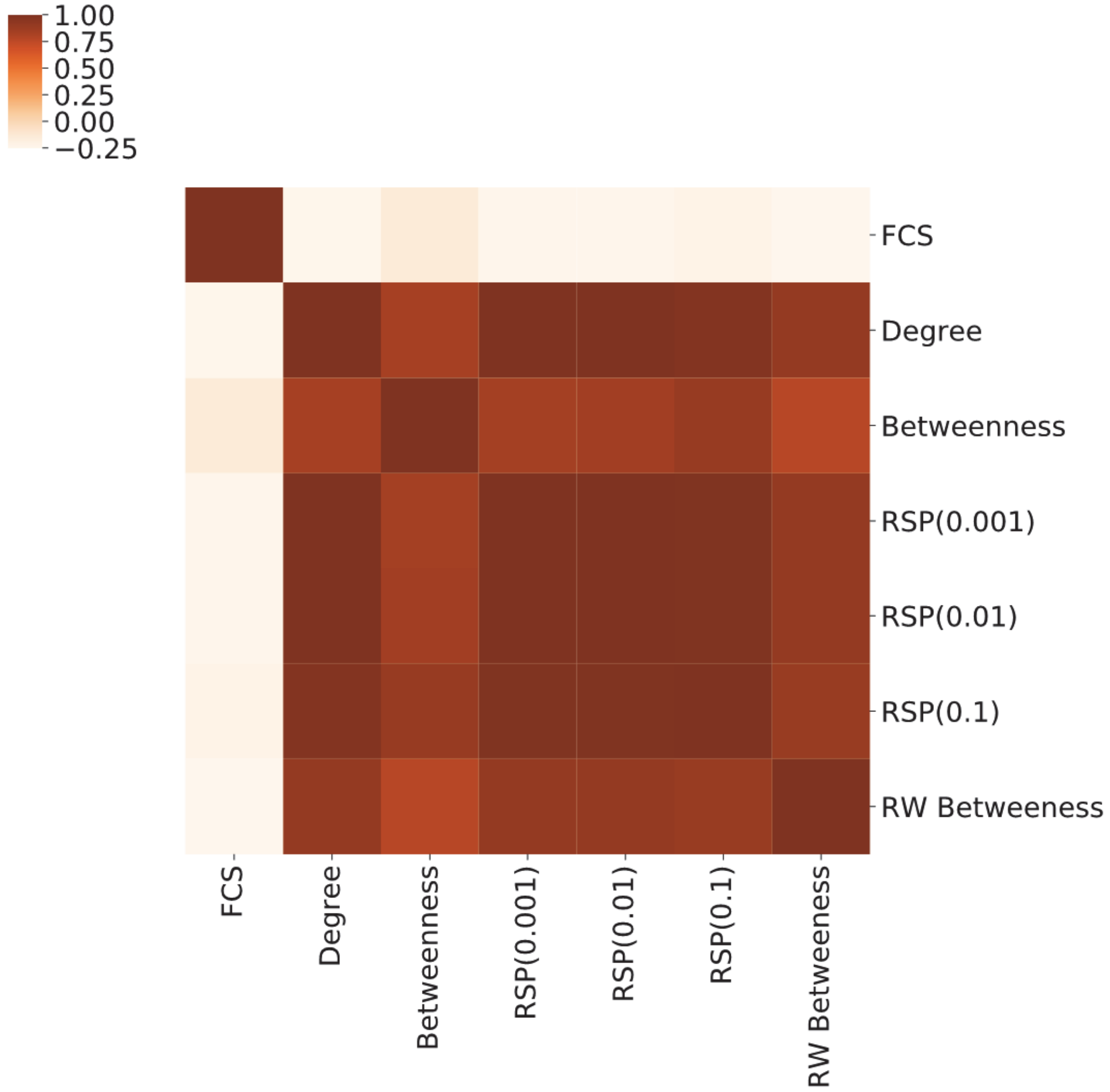

Supplementary Figure 3. Spearman's correlation of different topological measures: Flow Centrality Score (FCS), Degree, Random Walk betweenness (RW Between.), Randomized Shortest Paths with inverse temperature parameter x (RSP(x)) and shortest-path betweenness

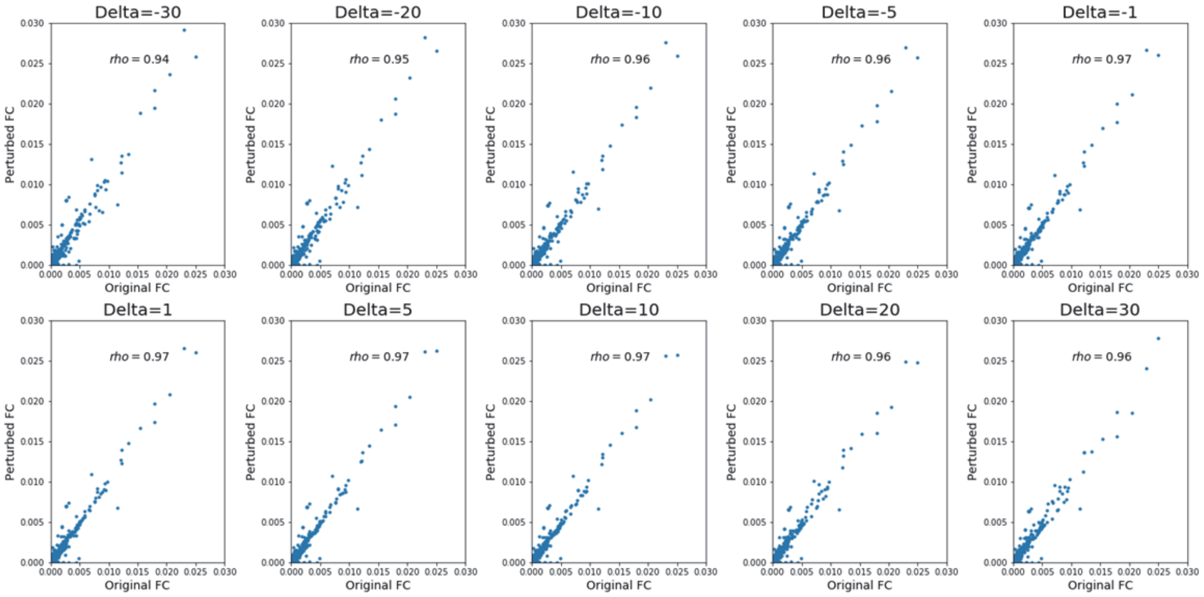

Supplementary Figure 4. Comparison of FC values of all genes for different perturbations of the DIAMOnD prioritization cutoff delta

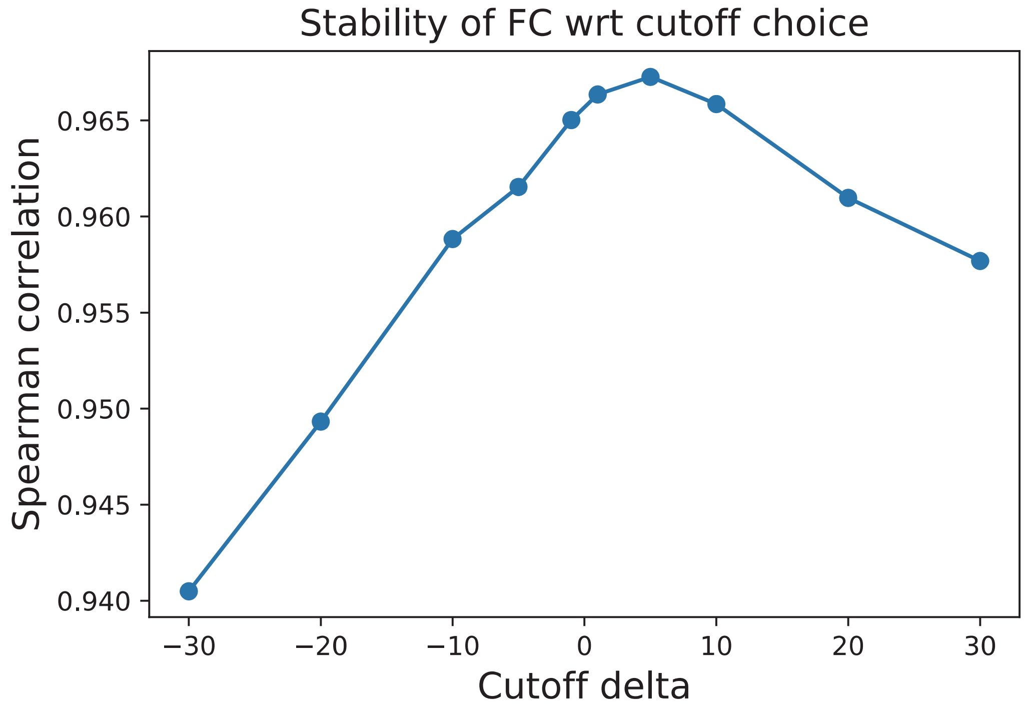

Supplementary Figure 5. Spearman's correlation coefficients between original FC values and perturbed FC values for different values of the cutoff delta

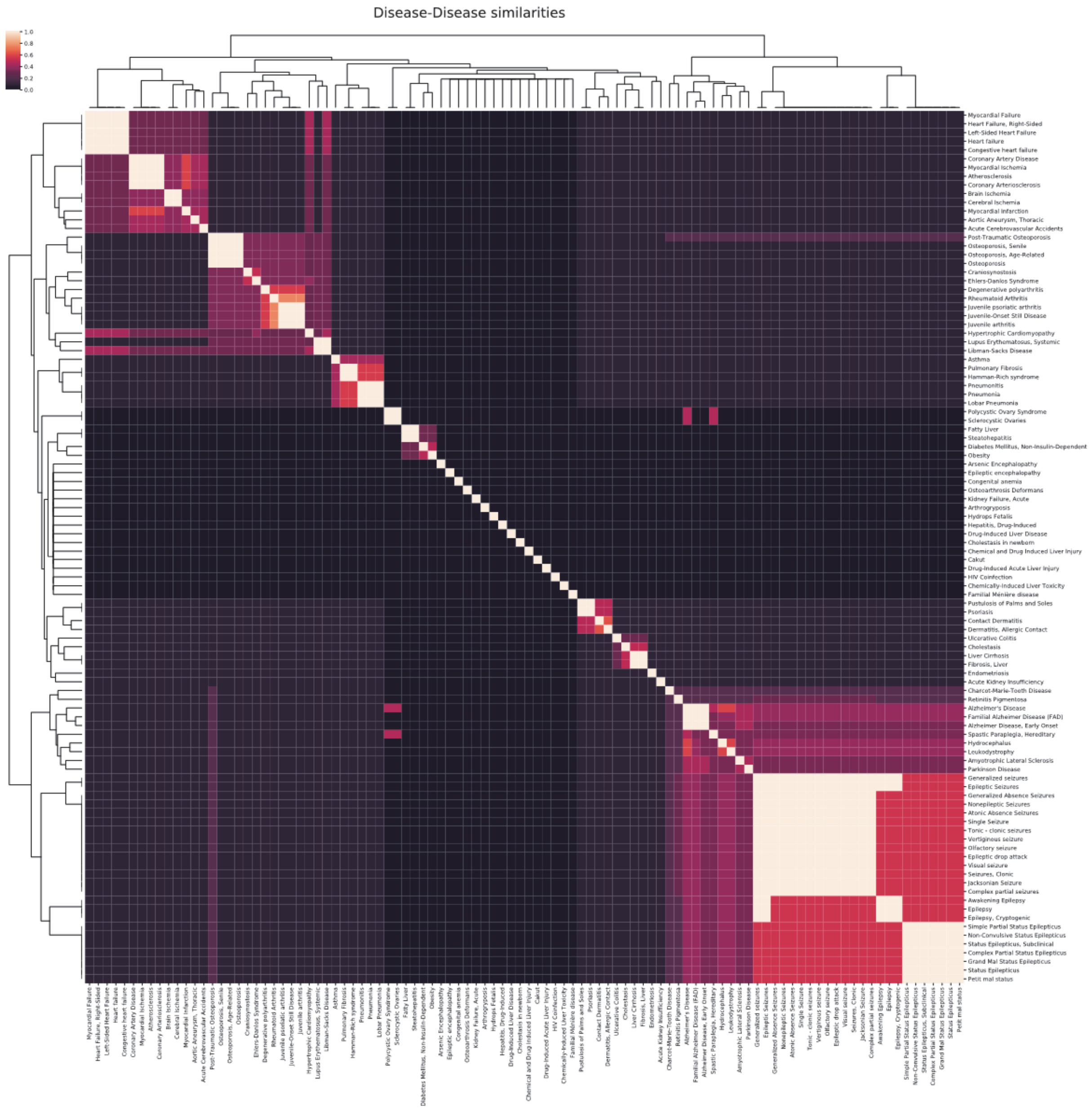

Supplementary Figure 6. Pairwise Resnik similarities between diseases and phenotypes extracted from DisGeNet, based on associated Disease Ontology terms.

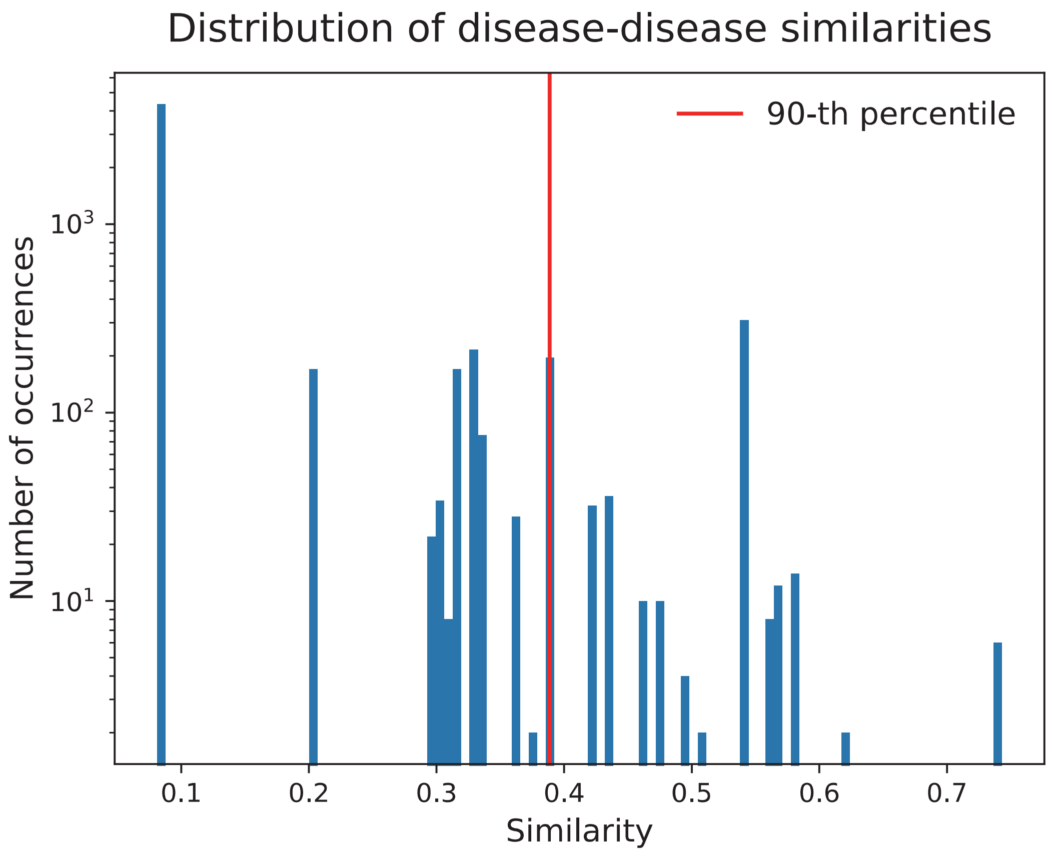

Supplementary Figure 7. Distribution of Resnik similarities between the diseases and phenotypes selected in DisGeNet. The red line indicates the 90th percentile of the distribution.

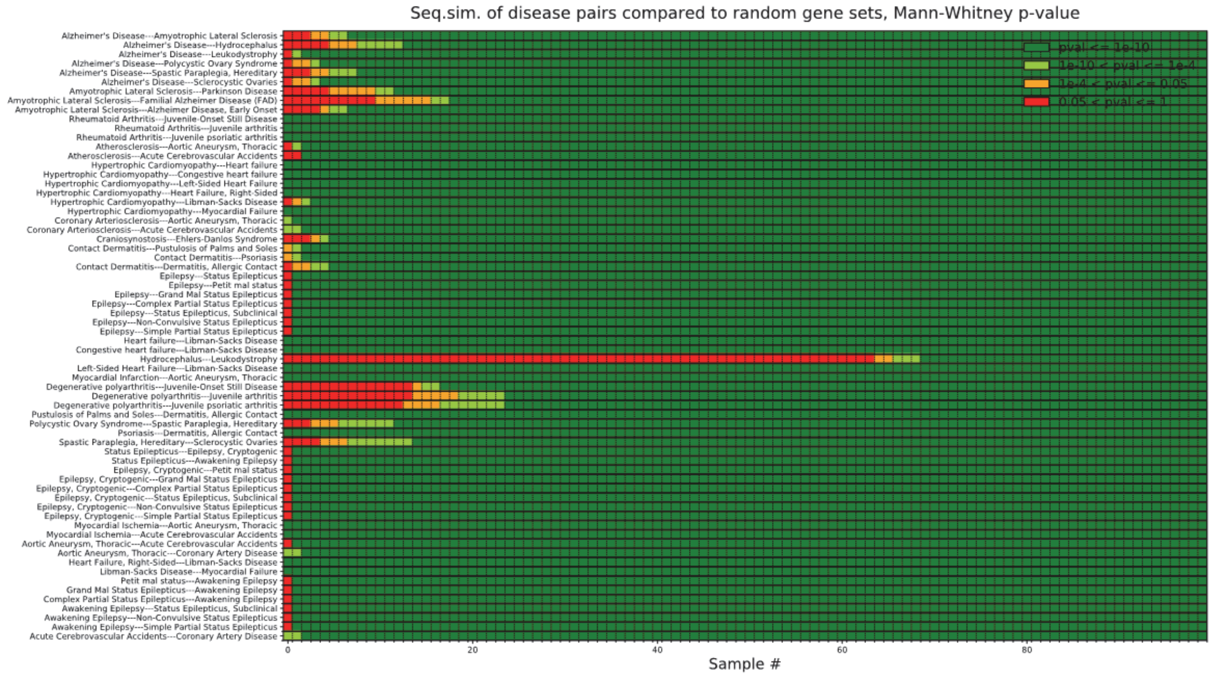

Supplementary Figure 8. Heatmap of the Mann-Whitney p-values resulting from the comparison FC paths of the original disease pair with the FC paths of the random pair, for each random pair. Each pair of random modules is obtained through a degree-preserved randomization of the original source and target disease modules.

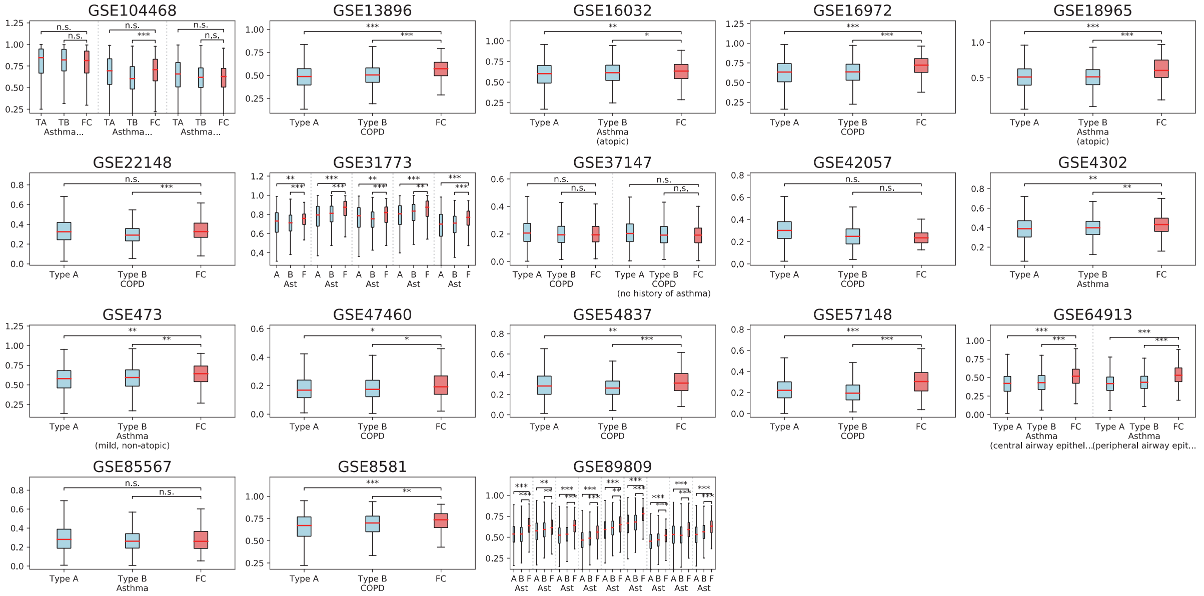

Supplementary Figure 9. Distribution of sequential coexpression values of Type A, Type B and FC paths for all the disease classes of the considered GEO datasets. In the boxplots, boxes indicate the quartiles, whiskers extend to an additional 1.5 * IQR interval, and the medians are highlighted in red. Asterisks indicate the degree of significance of the result, where ***, **, and

* respectively denote a p-value that is less than 1e-10, 1e-4 and 0.05, and ``n.s." stands for a non-significant result.

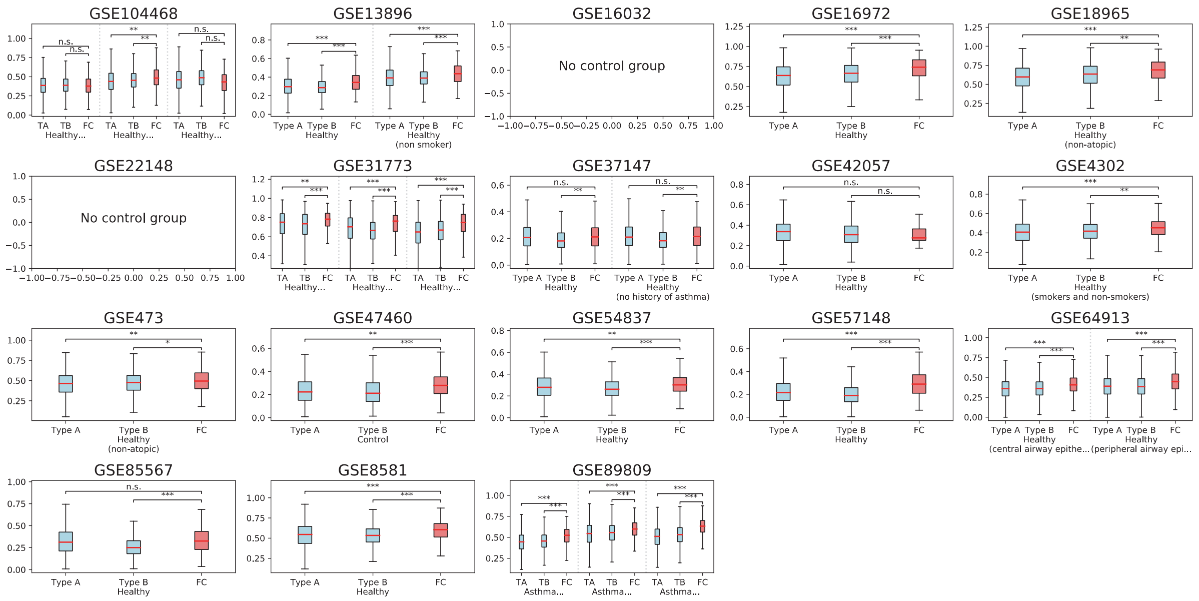

Supplementary Figure 10. Distribution of sequential coexpression values of Type A, Type B and FC paths for all the healthy classes of the considered GEO datasets. In the boxplots, boxes indicate the quartiles, whiskers extend to an additional 1.5 * IQR interval, and the medians are highlighted in red. Asterisks indicate the degree of significance of the result, where ***, **, and * respectively denote a p-value that is less than 1e-10, 1e-4 and 0.05, and ``n.s." stands for a non-significant result.
