# Supplementary material for: Discovering the genes mediating the interactions between chronic respiratory diseases in the human interactome
Source: Nat Commun. 2020 Feb 10;11:811. doi: 10.1038/s41467-020-14600-w (PMC7010776; doi:10.1038/s41467-020-14600-w)
Supplement: Supplementary file 3 — Description of Additional Supplementary Files [file 41467_2020_14600_MOESM3_ESM.pdf]

Description of Additional Supplementary Files

File Name: Supplementary Data 1
Description: Asthma seed genes and respective sources

File Name: Supplementary Data 2
Description: COPD seed genes and respective sources

File Name: Supplementary Data 3
Description: Overlapping genes between the asthma and COPD disease modules

File Name: Supplementary Data 4
Description: Flow centrality of genes when considering the asthma module as source and the COPD module as target

File Name: Supplementary Data 5
Description: List of related disease pairs extracted from DisGeNet

File Name: Supplementary Data 6
Description: List of GEO datasets considered in the analysis and their characteristics
